# Supplementary material for: Interferons and tuft cell numbers are bottlenecks for persistent murine norovirus infection
Source: PLoS Pathog. 2024 May 3;20(5):e1011961. doi: 10.1371/journal.ppat.1011961 (PMC11095769; doi:10.1371/journal.ppat.1011961)
Supplement: S1 Fig — (A) The genome copy:plaque forming unit (PFU) ratio for the 20 individual barcoded viruses was assessed in comparison to the relative proportion of each barcoded virus in the inoculum. (B,C) Cd300lf-/- mice (N = 5) were inoculated with CR6BC and stool viral shedding at 3 and 5dpi (B) and tissue viral levels at 5dpi (C) were assessed using qPCR. (D,E) Barcode richness of Cd300lf-/- stool (D) and tissues (E) was determined. (F) The proportion of mice (N = 16 total including WT and Ifnlr1-/- mice depicted in Fig 2) with each individual barcode detected at any time between 5-21dpi was compared to the level of that barcode in the inoculum. (G) The proportion of reads in the indicated tissues maintaining barcodes was assessed at 21dpi. Results were analyzed by Mann-Whitney test. ns, not significant. (DOCX) [file ppat.1011961.s001.docx]

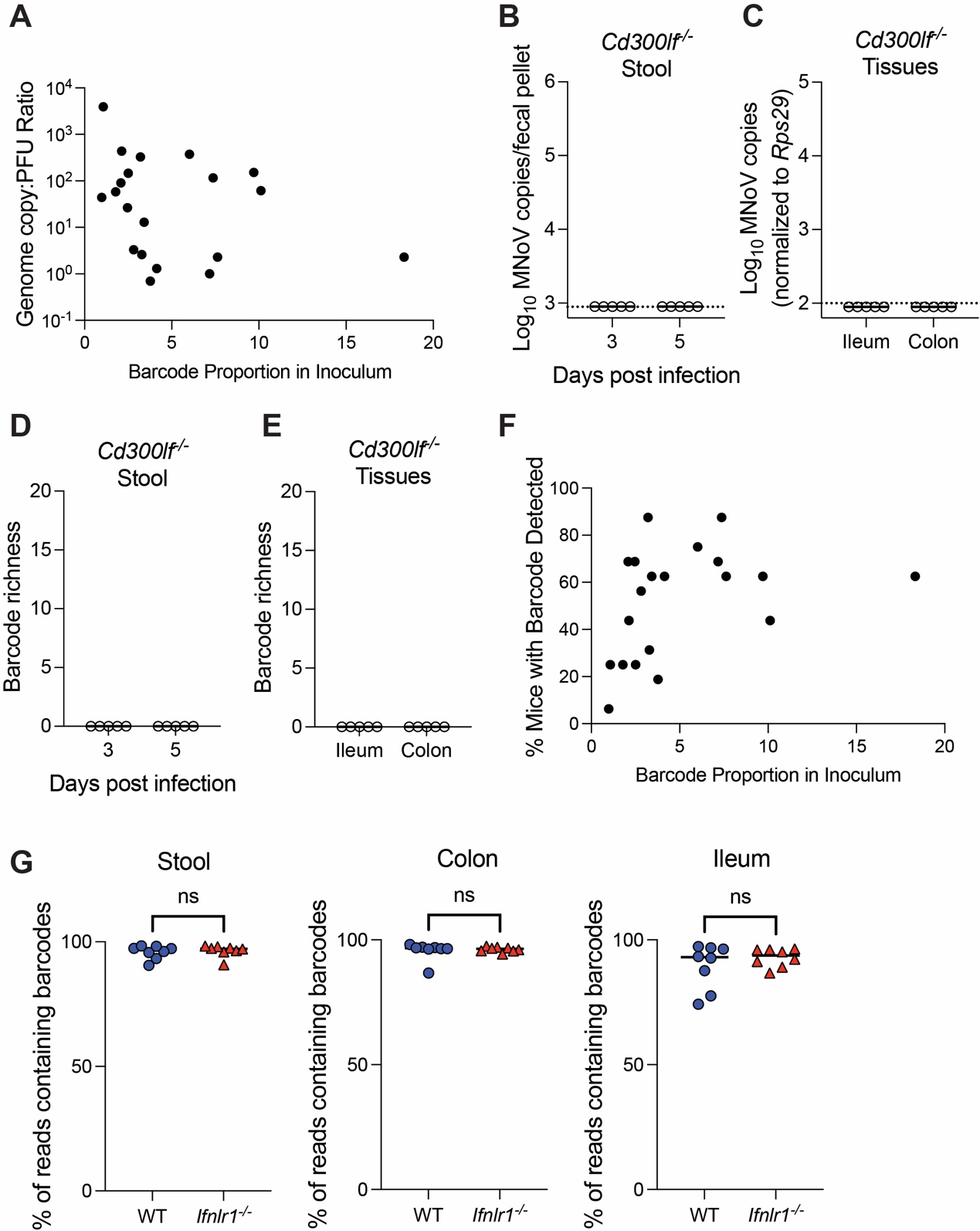


**S1 Fig: Barcoded CR6 (CR6^BC^) shows similar infectivity to CR6 and maintains barcodes *in vitro* and *in vivo.*** (**A**) The genome copy:plaque forming unit (PFU) ratio for the 20 individual barcoded viruses was assessed in comparison to the relative proportion of each barcoded virus in the inoculum. (**B,C**) *Cd300lf^-/-^* mice (N=5) were inoculated with CR6^BC^ and stool viral shedding at 3 and 5dpi (**B**) and tissue viral levels at 5dpi (**C**) were assessed using qPCR. **(D,E)** Barcode richness of *Cd300lf^-/-^* stool (**D**) and tissues (**E**) was determined. (**F**) The proportion of mice (N=16 total including WT and *Ifnlr1^-/-^* mice depicted in Figure 2) with each individual barcode detected at any time between 5-21dpi was compared to the level of that barcode in the inoculum. (**G**) The proportion of reads in the indicated tissues maintaining barcodes was assessed at 21dpi. Results were analyzed by Mann-Whitney test. ns, not significant.
