# Supplementary material for: Interferons and tuft cell numbers are bottlenecks for persistent murine norovirus infection
Source: PLoS Pathog. 2024 May 3;20(5):e1011961. doi: 10.1371/journal.ppat.1011961 (PMC11095769; doi:10.1371/journal.ppat.1011961)
Supplement: S1 Table — Sequences in red were random sequences used for the initial validation of the sequencing run and analysis; the last sequence (26) is the CR6 viral sequence with no barcode. (DOCX) [file ppat.1011961.s002.docx]

| **Barcode Number** | **Barcode Sequence** |
| --- | --- |
| 1 | GACGGAAATCGTTGGTAC |
| 2 | GACGGAAATTATTGGTAC |
| 3 | GACGGAACTGTCTGGTAC |
| 4 | GACGGAAGCACTTGGTAC |
| 5 | GACGGAAGTAGTTGGTAC |
| 6 | GACGGAATTTGTTGGTAC |
| 7 | GACGGACGCACCTGGTAC |
| 8 | GACGGAGACTGTTGGTAC |
| 9 | GACGGAGCTCTCTGGTAC |
| 10 | GACGGAGCTGTTTGGTAC |
| 11 | GACGGAGTCTCTTGGTAC |
| 12 | GACGGAGTTAGTTGGTAC |
| 13 | GACGGATCTACTTGGTAC |
| 14 | GACGGATGCGCTTGGTAC |
| 15 | GACGGATGCTATTGGTAC |
| 16 | GACGGATGTAGCTGGTAC |
| 17 | GACGGATGTGATTGGTAC |
| 18 | GACGGATGTGGTTGGTAC |
| 19 | GACGGATTCTACTGGTAC |
| 20 | GACGGATTTTCTTGGTAC |
| 21 | GACGGAAACCGTTGGTAC |
| 22 | GACGGAACCTTTTGGTAC |
| 23 | GACGGAAGTTGTTGGTAC |
| 24 | GACGGACTCGATTGGTAC |
| 25 | GACGGAGTCAATTGGTAC |
| 26 | GACGGATGGTAC |

**S1 Table:** List of the sequences containing 6 nucleotide barcodes (underlined) used in the study. Sequences in red were random sequences used for the initial validation of the sequencing run and analysis; the last sequence (26) is the CR6 viral sequence with no barcode.
