# Supplementary material for: Interferons and tuft cell numbers are bottlenecks for persistent murine norovirus infection
Source: PLoS Pathog. 2024 May 3;20(5):e1011961. doi: 10.1371/journal.ppat.1011961 (PMC11095769; doi:10.1371/journal.ppat.1011961)
Supplement: Attachment — Submitted filename: Response to Reviewers_v2.docx [file ppat.1011961.s012.docx]

**Response to Reviewers for "Interferons and tuft cell numbers are bottlenecks for persistent murine norovirus infection" (Manuscript ID - PPATHOGENS-D-24-00060)**

Reviewer comments are in **black**; author responses are in **blue**

Reviewer #1: This report by Aggarwal and colleagues, seeks to understand the consequences of host innate immunity on norovirus population dynamics. Using the murine norovirus (MNV) model system, the authors generate a pool of 20 different MNV clones each containing a genetic barcode by inserting 6-nucleotoide sequences at the 3’ end of the viral NS4 coding sequence. Using this pool of barcoded MNV the authors find that persistent infection involves a series of reinfection events and genetic richness decreased over time, with IFN lambda receptor knockout mice, viral richness and replication in the intestines was increased. Increasing host tuft cell numbers by IL-4 treatment increased barcode richness suggests tuft cell numbers are a bottle neck during MNV infection. Finally, Stat1 knockout mice showed increased viral diversity at extraintestinal sites indicating different interferons as critical bottlenecks at tissue types, and that disseminated splenic viruses were distinctly barcoded from intestinal viruses. This greater understanding of how host cell immunity influences norovirus population dynamics is original and of high importance to researchers in this field and likely of a broader interest to those studying related viruses (in particular human norovirus).
Generally the manuscript is very well written and described, the methodology is overall rigorous and experiments well controlled. The authors conclusions I believe are supported by the evidence provided. There are some additional data that would be informative to the study, in addition to some minor changes to help readability.

We thank the reviewer for their thoughtful comments and are delighted that they appreciate our study.

Reviewer #1: 1. The “barcodes” used are inserted after nucleotide 2601 which lies at the C-terminal end of NS4. It would be informative to provide additional information on the barcodes used and how they alter amino acid sequence, where they lie in respect to the NS4NS5 junction and if they alter processing of the polyprotein. Additional methodological information will allow other researcher studying MNV and other viruses to use a similar approach.

The cleavage site between NS4 (p18) and NS5 (VPg) occurs at ^870^E/G^871^ (Sosnovtsev et al., 2006), with the barcode insertion of 2 amino acids occurring between amino acids 865 and 866. We have now included this information more clearly in the manuscript in our Materials & Methods section. While we did not directly assess whether barcode insertion modified polyprotein processing in our study, prior insertion of a FLAG tag at this site yielded tagged NS4 of the expected molecular mass, suggesting that polyprotein processing is not disrupted by insertions at this site.

We have also now included a table of the 20 barcode sequences used in our study (Supplementary Table 1).

2. The authors show CR6BC is sequence stable in vitro for 72 hours however several of the mice experimental groups are up to 21 days post infection. Are the barcodes maintained without change or loss in vitro or in vivo up to 3 weeks. Likewise, is there any difference or variations in the replication competency of individual barcoded viruses? Differences or reduced replication competency many be expected since the original transposon mutagenesis paper where this insertion was characterised (Thorn et al 2012) suggested insertions after nucleotide 2600 cause ~2-log reduction in viral replication.

In Fig 2C, we show that the barcodes are maintained in stool over the course of even 21 days of infection. We have also now analyzed our barcode sequencing data in tissues at day 21, and similarly found that the majority of reads in tissues contain barcodes. Those reads lacking barcodes also do not have detectable WT virus, and so these reads may represent either virus with mutated barcodes and/or sequencing errors. These data are now included as Supplemental Figure 1G.

In the Thorne et al. study, while the authors observed a substantial reduction in titer with the insertion of a 5 aa sequence at the NS4 site (Fig 6 of that paper), this site does tolerate insertion of an 8 aa FLAG tag without loss of viral replication efficiency (Fig 7B of that paper). While we did not characterize the replication of individual barcoded viruses, we did observe that the pool of viruses with 2 aa barcode insertions was able to replicate similarly to WT virus both *in vitro* and *in vivo* (Fig 1B and 1E of our study).

3. In Figure 1D, it was unclear why the relative proportions of sequence reads for each barcoded virus are different when the same number of PFU were pooled to generated CR6BC. Does this represent bias in the sequence data or different PFU:particle ratios?

We have now analyzed the genome copy (GC):PFU ratio for individual barcoded viruses, and found wide variation in these ratios, which we have also previously observed among WT CR6 stocks. While there is no clear relationship between GC:PFU ratio and proportion of each barcoded virus in the inoculum (please see graph below), this variation could certainly be a contributor to the uneven representation of each barcode in the inoculum, a point we have now added to the text. We have also now included the graph below as Supplemental Figure 1A.

4. Lines 219:220: Here the manuscript says “distinct barcodes were observed in each mouse, indicated that this low number was not secondary to poor viability of some clones”. It is hard for the reader to conclude this from the data presented in Fig 2B, it could be that one or two clones are poorly viable even if different barcodes were prevalent in each mouse/sample. Were there any statistically significant differences in the barcodes represented which may suggested poorer viability.

We have now included new Supplementary Tables 2, 3, and 4 which explicitly show the representation of individual barcodes within each mouse. We have also compared the representation of these barcodes in the inoculum with their representation in mice (using the 16 mice in Figure 2), which shows a non-statistically significant correlation between detection in mice of each barcode and initial levels in the inoculum. This correlation is now provided as Supplementary Figure 1F.

While these data provide important new details for the reader about the specific barcodes tested, we would like to emphasize that they also highlight that distinct individual barcoded viruses establish infection in mice in different combinations and with varying barcode richness depending upon the genetic background and treatment status of the mice.

To the reviewer’s point, there may be poor viability of a few of the clones which is not something we have explicitly tested, so we have also adjusted the wording to “secondary to poor viability of numerous specific clones” to better reflect that MOST clones are well-detected across many mice.

5. The choice of colours and symbols for some figures (e.g., Figure 2) makes it harder to interpret for some readers with types of colour blindness. Could more distinct colours and/or symbols be used to make all the figures for accessible.

We fully agree with the reviewer’s comment and have adjusted our colors and symbols in Figures 2, 4, and 5 to be more accessible for colorblind readers.

6. Figure 5: Here the authors have conducted a thorough statistical comparison, however, is a comparison of all groups to each other required. Could the figure be simplified by only presenting key statistical comparisons or only highlighting the significant comparisons.

As mentioned above, we have now changed the colors and symbols in this figure, and have also now removed the “ns” comparisons to make the overall figure and the statistical comparisons easier for the reader to assess.

Other minor comments
7. Line 94: Define VP-SFM

8. Line 141: For consistency uL not ul
9. Line 153: Subscript 4 i.e., MgSO4
10. Line 184: NS4 not p18

We have now modified our manuscript to revise all of these points.

Reviewer #2: In this well written and straightforward manuscript, the authors describe their creation of a panel of 20 barcoded viruses using the persistent murine norovirus strain, CR6. After measuring in vitro replication efficiency, the authors infected this virus pool into wild type and transgenic mice and quantified these viruses in intestinal tissue, the spleen and the stool. The barcodes allowed the authors to also evaluate virus populations in these tissues. The major conclusions drawn by the authors from their results include: 1) virus is predominantly shed in stool comes from viruses that are infecting the colon, 2) that viral barcode richness decreases over time in all tissues and in all mice, regardless of immune status, 3) but that richness is increased in IFN-lambda mice compared to all others and that this increase in richness is due to improved viral replication; 4) increases in the number of tuft cells (induced by IL-4 treatment) increases richness, likely also due to improved replication resulting from the increased availability of tuft cells; 5) loss of IFN-I signaling increased viral diversity in extraintestinal sites indicating that IFN-I is responsible for limiting spread of MNV; 6) that intestinal viruses were different from extraintestinal viruses. The data presented in the manuscript provides important insight into norovirus replication, shedding and dissemination and the tool of barcoded viruses created to do these studies will likely prove to be extremely valuable in discerning other aspects of norovirus pathogenesis and replication. While the results presented in the manuscript are significant, there are some concerns which should be addressed to strengthen the results and improve the clarity of the overall conclusions.

We appreciate the reviewer’s enthusiasm for our work and thank the reviewer for their helpful comments.

Reviewer #2:

1. Lines 189-190. The barcoded viruses are not in equivalent numbers based on in vitro work. While no single virus makes up more than 15% of the population it appears some viruses make up less than 1% of the pool. When in vitro work was repeated, was this stratification of population consistent? Did some viruses always become predominant? Was it always the same viruses? Do the authors have an explanation for why, if equal PFUs were added to the pool, some barcodes drop below 1% of the population? Does this indicate that some of the viruses in the pool have growth defects which could impact interpretation of in vivo results?

Indeed when we assessed the portions of our inoculum that are made up by the different barcodes, we found a range between 0.7-13.6% for one viral pool and 1.0-18.5% for the second. We have now provided these raw read data for the barcode inocula as well as for our analyses of different stool and tissue samples in Supplemental Tables 2, 3, and 4. As discussed above in response to Reviewer #1’s question #3, we analyzed GC:PFU ratios and found these were quite distinct between individual barcoded viruses, raising one possibility for differential performance of individual barcoded stocks. While some barcodes were consistently represented at low levels in the inoculum (e.g. barcode #11) and others at high proportions (e.g. barcode #12), both were detected in multiple distinct mice, suggesting that all individual barcoded viruses were viable *in vivo*. Distinct barcode combinations were represented in each individual WT mouse, for example, and the proportions of represented barcodes are quite distinct between treatment or genotype groups, and thus while our individual barcoded viral stocks did perform variably, we are not concerned that this is leading to misinterpretation of our *in vivo* results.

It is stated on line 219 that the in vivo data indicates there is no differences in clone viability, but that specific barcode data is not provided (Fig 2).

Reviewer #1 raised a similar concern in their question #4; we have now adjusted our text and added an additional supplemental figure to clarify that most of our barcoded viruses were well-represented across mouse lines.

2. Lines 219-220. Since the authors possess data showing the distinct barcodes at each site, including that data in the supplemental section would be helpful and also possibly provide insight into any issues with in vivo replication of specific viral barcodes.

We fully agree and have now provided all of the individual barcode read information in Supplemental Tables 2, 3, and 4.

3. Lines 231-233. Which barcodes are lost during persistent infection? Is it consistent between mice? Including a table with this data in the supplemental section would be helpful.

We have now provided these data as Supplemental Table 2. We did not observe any clear pattern in which barcodes were lost between initial detection at 5dpi at subsequent persistent timepoints of 14 and 21dpi. This varied depending upon the barcodes initially establishing infection and distinct mice exhibited distinct “drop-out” of different barcodes over the course of infection.

4. Lines 276-277. This conclusion takes into consideration only the likelihood test mentioned immediately prior while ignoring the rest of the data presented in this section showing that the method used cannot sufficiently determine if viruses shed in stool comes from ileal virus or colonic virus. The statement should be revised to accurately reflect all the data presented in this section.

We have now softened our conclusion by including the word “likely” as well as broadened our final statement for this section to include “and also indicate that throughout infection, the majority of virus is shared between both small and large intestinal tissues.”

5. Lines 291-304. The authors state that tufts cells are the reservoir for persistent virus infection, yet the data displayed in this section only examines 5 dpi (previously indicated by the authors as the “acute timepoint”. How did the increase in tufts cells alter diversity at the 21 dpi, persistent timepoint? Also, it is not stated how the authors validated that IL-4 treatment increased the abundance of tufts cells and how large was that increase. If not possible to perform, the authors should include typical tuft cell number increases.

We have now included data (new Figure 4A and 4B) validating the expected IL-4 treatment-associated increase in tuft cell abundance by performing qPCR for tuft cell marker genes *Dclk1* and *Cd300lf* (also the MNoV receptor). We found that at 5 dpi these markers were significantly increased in both WT and *Ifnlr1^-/-^* mice treated with IL-4 in ileum and colon samples.

We did not evaluate the effects of IL-4 treatment beyond 5 dpi, as the effects of IL-4 treatment are transient in boosting tuft cell numbers. We have now clarified in the text that tuft cells are “the exclusive cells infected by CR6 in the intestinal epithelium in the acute setting and also provide an immune-privileged niche for persistent viral infection”.

6. Lines 344-346. As indicated previously by the authors, barcode richness appears to be tied to viral replication. Therefore, do the Type I and Type III IFNs actually and directly control viral richness as the authors conclude? Or do they rather have an indirect effect related to their ability to regulate viral replication and dissemination? Acknowledgement and expansion of this point in the manuscript would be helpful.

We fully agree that the likely mechanism by which barcode richness is modulated is via modulation of viral replication/levels; we have now better incorporated that understanding into this sentence by modifying it to say “combinatorial roles for type I and III IFNs, with possible contribution from type II IFN, in limiting extraintestinal viral replication and thereby diversity.”

We have also added a statement into our discussion emphasizing this point further: “the viral diversity bottlenecks observed in WT mice, consistent with their roles in regulating viral replication and dissemination.”

7. Lines 359-373 . Why did the authors not include ileal barcodes in their comparison with the spleen? Given that the ileum contains the majority of Pyers Patches and the connection of PPs to the MLNs which drain to other lymphatic tissues, could there be a correlation between ileal replication and migration to the spleen? This data is very related to the conclusions the authors are trying to draw. Even including data from WT mice would be helpful (in the case that ileal tissue wasn’t collected for the transgenic mice).

We fully agree that in retrospect, analysis of ileal tissue in our study of viral dissemination (Figures 5 and 6) would have been quite helpful to our understanding of initial infection events and how they contribute to spread, and indeed direct analysis of PPs versus other ileal tissue and MLNs would have also been beneficial. Unfortunately, we did not analyze these tissues for these studies. Further, our studies in WT mice did not show spread to spleen and we did not collect matched splenic tissue in our persistent infection experiments when studying ileal infection (Figures 2, 3, and 4). We are enthusiastic to incorporate the reviewer’s excellent suggestion into our future studies using barcoded viruses.

Reviewer #3: The manuscript titled “Interferons and tuft cell numbers are bottlenecks for persistent murine norovirus infection” submitted for publication by Aggarwal et al describes the use of a barcode-based technology to study norovirus pathogenesis in a murine model. This paper addresses unanswered questions surrounding host factors, such as epithelial cell types and immune signaling pathways that drive norovirus population dynamics. The manuscript is extremely well written, and the experiments are well designed, executed, and validated with controls and statistics including upfront validation of the bar-coded viral strains compared to wild type strains. In general, the conclusions are appropriate based on the data presented. The bar code technology has been used with other viral systems, and the authors capitalize on its availability to adapt it to the murine norovirus model. New discoveries include the concept that IFNg is a signaling bottleneck for intestinal murine norovirus replication, the colon as the major contributor to stool shedding of murine norovirus, the presence of the tuft cell as a bottleneck for intestinal murine norovirus replication, and the role of Type I IFN and not intestinal viral replication in limiting extraintestinal spread of murine norovirus. These findings are interesting and provide substantial insight into knowledge of murine norovirus replication, extra-intestinal spread, and pathogenesis that warrants publication in PLOS Pathogen. The discussion of the paper seems a bit lacking on integrating these findings into the bigger picture of the pathogenesis of other murine norovirus strains (ie are these findings only specific to the CR6 strain) and more importantly, if any of the findings relate to human norovirus pathogenesis.

We thank the reviewer for their considerate comments; we have endeavored to carefully address these below.

Reviewer #3: • The percentage of barcode recovery in Fig 1D seems to vary from close to 0% all the way to about 12%. If each barcode is used in equal proportions, how is this difference explained?

This concern was shared by the other two Reviewers and is further discussed above; we have now included raw data for the reads detected from inocula in Supplemental Tables, and included analysis of genome copy:PFU ratios in Supplemental Figure 1.

• Re the conclusion in lines 266-268, What is the limit of detection of the bar codes. Is it possible that bar coded viruses are contributing to the overall viral load but are below the limit of detection of the approach?

We have now made the results of our barcode sequencing much more transparent with the inclusion of Supplemental Tables 2, 3, and 4. The limit of detection for the barcodes was 10 reads, which was derived from within-sequencing-run analysis of naïve mice to assess for any nonspecific detection/PCR artifacts. This information has been added to our methods section.

It is possible that there may be barcoded viruses that are contributing to viral load at low levels below our limit of detection; we have more clearly included this as a caveat of our study in the discussion with the following statement, “We also cannot exclude the possibility of barcodes below the limit of detection in our assay contributing to viral replication.”

• There seems to be variability between figures 2 and figures 4 and 5 in terms of the barcode richness in the controls. Is this difference statistically significant and what is the relevance of this variability to the findings and conclusions?

We directly compared 5 dpi Figure 2 and Figure 5 WT control barcode richness data in stool and colon samples and found no significant differences.

Reviewer #3: • In figure 1, it would improve the flow of the paper to have the proportion of barcoded viruses in the stool and tissue to go along with Fig 1E/F. Splitting into two figures with one on the BV2 cells and one on the mouse model would be good.

We appreciate the reviewer’s suggestion. Due to Figure 2 already having quite a few panels and the distinct mice used for evaluating the proportions of barcoded viruses, we have elected to maintain the original configuration of the figures. We have now included Supplemental Tables to further clarify all the barcoded information.

• In lines 220-222, the conclusion seems overstated. It could just be random chance that specific bar codes were observed rather that the fact that a limited number of unique viruses could establish/maintain infection.

While the barcodes we observed in each mouse were unique combinations (now clearly shown in Supplemental Table 2), the observation that in some mice (e.g. *Ifnlr1-/-* and *Stat1-/-*), all or nearly all barcodes can be observed while in WT these are not seen under the same conditions, supports that WT mice experience bottlenecks that are reflected in the limited barcode richness.

• In Fig. 2, it is hard to tell the triangles apart. The use of a different shape/color would improve the readability of this figure.

We agree with the reviewer’s comment and have now changed both the color and shape of points in the figure to improve readability.

• In Fig 2C, the data is shown as MNoV copies per fecal pellet but there is no description in the materials and methods as to how this was collected. A random sample from the cage versus one taken directly from the mouse at the same time might have totally different levels of virus.

We have now clarified our Materials & Methods section to specify that all stool samples were collected directly from individual mice. Further, the mice were singly-housed upon infection, a detail that is also now included for greater clarity.

• In lines 263-264, how do the authors imagine that colonic infection contributes to ileal infection? Does CR6 cause viremia?

We would speculate that coprophagy of shed virus would contribute to re-infection of the ileum in this mechanism. We have now clarified the statement to include this point: “Whether ileal infection contributes to colonic infection or vice-versa, wherein re-infection of ileal tissue could occur secondary to coprophagy of colon-derived shed virus, is unclear.”

• Re the conclusion in lines 276-277, how many tuft cells are present in the mouse colon compared to the small intestine that would support this conclusion?

There are broadly similar numbers of tuft cells in ileum and colon tissues at baseline, with similar detectable expression of tuft cell marker *Dclk1* expression now provided in new Figure 4A.

• In lines 407-410, it should also be considered that tuft cells in the colon preferentially express factors that are important for viral binding and entry.

We agree with the reviewer’s point, and indeed see some modest increased trend in *Cd300lf* expression in the colon compared to ileum (new Figure 4B). We have now modified the statement to include this point: “differential capacity to be infected or to produce or package virus”.

• Discussion of the following reference should be included: Sharon AJ, Filyk HA, Fonseca NM, Simister RL, Filler RB, Yuen W, Hardman BK, Robinson HG, Seo JH, Rocha-Pereira J, Welch I, Neyts J, Wilen CB, Crowe SA, Osborne LC. Restriction of Viral Replication, Rather than T Cell Immunopathology, Drives Lethality in Murine Norovirus CR6-Infected STAT1-Deficient Mice. J Virol. 2022 Mar 23;96(6):e0206521. doi: 10.1128/jvi.02065-21. Epub 2022 Feb 2. PMID: 35107369; PMCID: PMC8941907.

We agree; this reference has now been added in the text in relation to the first description of STAT1 activity.

• Lines 434-436, limits of detection should still be considered here as a limitation.

We have now included limit of detection of our barcodes as a limitation of our study.
